# Data of chemical analysis and electrical properties of SnO_2_-TiO_2_ composite nanofibers

**DOI:** 10.1016/j.dib.2018.03.110

**Published:** 2018-03-28

**Authors:** Zinab H. Bakr, Qamar Wali, Jamil Ismail, Naveen Kumar Elumalai, Ashraf Uddin, Rajan Jose

**Affiliations:** a Nanostructured Renewable Energy Materials Laboratory, Faculty of Industrial Science & Technology, Universiti Malaysia Pahang, Gambang, 26300 Kuantan, Malaysia; b Physics Department, Faculty of Science, Assiut University, Assiut 71516, Egypt; c Materials Research Laboratory, Department of Physics, University of Peshawar, Peshawar 25120, Pakistan; d School of Photovoltaics and Renewable Energy Engineering, University of New South Wales, Sydney 2052, Australia

## Abstract

In this data article, we provide energy dispersive X-ray spectroscopy (EDX) spectra of the electrospun composite (SnO_2_-TiO_2_) nanowires with the elemental values measured in atomic and weight%. The linear sweep voltammetry data of composite and its component nanofibers are provided. The data collected in this article is directly related to our research article “Synergistic combination of electronic and electrical properties of SnO_2_ and TiO_2_ in a single SnO_2_-TiO_2_ composite nanowire for dye-sensitized solar cells” [1].

**Specifications table**

**Table:** 

Subject area	Materials science
More specific subject area	Composite nanofibers
Type of data	Table and figures
How data was acquired	potentiostat-galvanostat (Autolab PGSTAT30, Eco Chemie B.V., The Netherlands) and energy dispersive X-ray measurements (EDX-720, Shimadzu, Japan)
Data format	analyzed
Experimental factors	50 mg powder samples were pressed into pellets of thickness ~ 1 mm and diameter ~8 mm using a hydraulic press at 5 t for 30 sec.
Experimental features	forward bias range: up to 5 V for linear sweep voltammetry measurement
Data source location	Universiti Malaysia Pahang, Gambang, 26300, Kuantan, Malaysia
Data accessibility	Synergistic combination of electronic and electrical properties of SnO_2_ and TiO_2_ in a single SnO_2_-TiO_2_ composite nanofibers for dye-sensitized solar cells

**Value of the data**

• Chemical analysis of composite nanowires using EDX further supports our interpretation homogenous structure of composite nanowire.
• Linear sweep voltammetry curves presented in this article for nanowires materials would be useful for insight of electrical properties behavior.
• The data obtained can be used in investigating the electrical conductivity of the metals oxide relating to its nature

## 1 Data

**Fig. 1:**
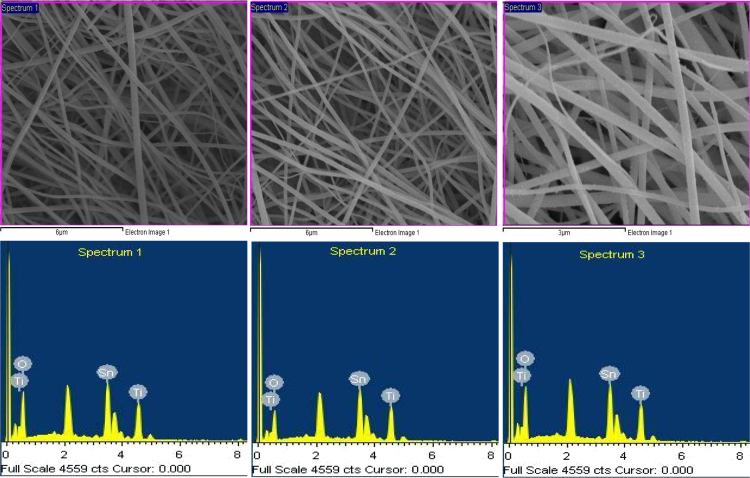
EDX analysis of the electrospun SnO_2_–TiO_2_ CNFs composite from three different selected areas.

**Fig. 2:**
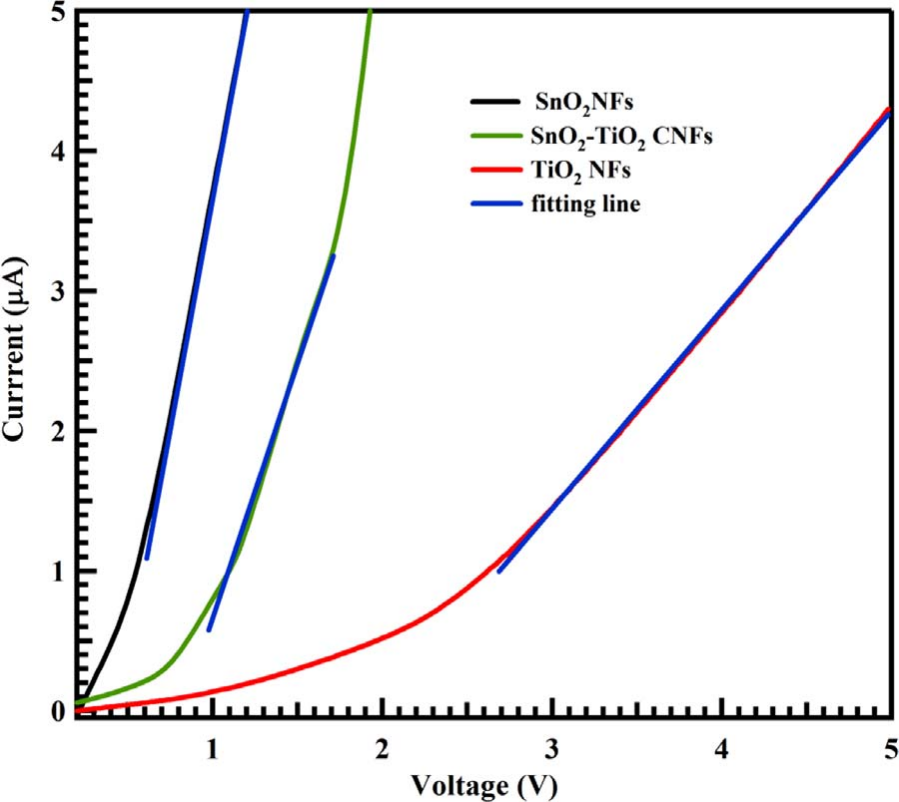
Linear sweep voltammetry of TiO_2_ NFs, SnO_2_ NFs and SnO_2_-TiO_2_ CNFs.

In order to identify the chemical analysis of SnO_2_-TiO_2_ composite nanofibers (CNFs) synthesized by electrospinning technique [1], EXD analysis was carried out. Three areas of these nanofibers were investigated as shown in Fig. 1. Details of the elemental values of Sn, Ti and O measured in atomic and weight % are listed in Table 1. The electrical properties characterization of the CNFs and counterparts are shown in Fig. 2.

**Table 1:** EDX weight rations of electrospun (SnO_2_ –TiO_2_) CNFs using three spectrums focused three distinct areas.

SnO_2_-TiO_2_ CNFs	**Tin (Sn)**		**Titanium (Ti)**		**Oxygen (O)**	
	**Wt. (%)**	**At. (%)**	**Wt. (%)**	**At. %**	**Wt. (%)**	**At. (%)**
Spectrum 1	39.10	9.67	17.48	10.71	43.41	79.62
Spectrum 2	42.91	11.67	19.99	13.48	37.10	74.85
Spectrum 3	37.05	8.86	17.37	10.29	45.58	80.85

## 2 Experimental design, materials and methods

The chemical composition of the nanofibers was determined by EDX. The three EDX spectrums of the calcined nanofibers at 500 °C, shown in Fig. 1 with the corresponding peaks, indicate the presence of Sn, Ti and O elements. In spectrum 1, the quantity of Sn, Ti and O were 9.67, 10.71 and 79.62, respectively, while in spectrum 2, the values were (11.67, 13.48 and 74.85 measured in atomic % for Sn, Ti and O, respectively). Details of the three EDX spectra of the electrospun (SnO_2_-TiO_2_) NFs values measured in atomic and weight % are listed in Table 1. Fig. 2 shown the dependence of the applied voltage for CNFs and counterparts measured by linear sweep voltammetry method. SnO_2_ shown sharp increase compared to TiO_2_. While CNFs is midway. Therefore, CNFs has conductivity midway the counterparts.
