# Supplementary material for: Data of chemical analysis and electrical properties of SnO2-TiO2 composite nanofibers
Source: Data Brief. 2018 Mar 28;18:860–3. doi: 10.1016/j.dib.2018.03.110 (PMC5996404; doi:10.1016/j.dib.2018.03.110)
Supplement: Supplementary file 1 — Supplementary material [file mmc1.docx]

Conflict of Interest

The authors declare no conflict of interest.
